# Stress Hyperglycemia and Outcome of Non-diabetic Patients After Acute Ischemic Stroke

**DOI:** 10.3389/fneur.2019.01003

**Published:** 2019-09-18

**Authors:** Bihong Zhu, Yuesong Pan, Jing Jing, Xia Meng, Xingquan Zhao, Liping Liu, Yilong Wang, Yongjun Wang, Zhimin Wang

**Affiliations:** ^1^Department of Neurology, Taizhou First People's Hospital, Affiliated Huangyan Hospital of Wenzhou Medical University, Taizhou, China; ^2^Department of Neurology, Beijing Tiantan Hospital, Capital Medical University, Beijing, China; ^3^China National Clinical Research Center for Neurological Diseases, Beijing, China

**Keywords:** diabetes mellitus, stress hyperglycemia, outcome, acute ischemic stroke, recurrent stroke

## Abstract

**Background and Purpose:** Stress hyperglycemia is relative hyperglycemia after suffering an acute illness such as stroke, even without preexisting diabetes. Our study aimed to determine the relationship between stress hyperglycemia and outcome of non-diabetic patients with acute ischemic stroke.

**Methods:** Data were derived from the ACROSS-China (Abnormal Glucose Regulation in Patients with Acute Stroke across China) registry. Patients with ischemic stroke but without a history of diabetes mellitus were included in this analysis. Stress hyperglycemia was measured by glucose-to-glycated hemoglobin (HbA1c) ratio which was calculated by fasting plasma glucose (FPG) divided by HbA1c. We investigated the association between quartiles of glucose-to-HbA1c ratio and stroke recurrence and all-cause death at 12 months after stroke onset.

**Results:** We included a total of 999 patients, among which there were 105 (10.9%) recurrent strokes and 76 (7.6%) deaths at 12 months. Using the lowest quartile of glucose-to-HbA1c ratio as the reference, patients with the highest quartile were associated with an increased risk of stroke recurrence [16.0 vs. 8.3%; adj.hazards ratio (HR) = 2.19, 95% confidence interval (CI): 1.26–3.83] and death (13.0 vs. 4.3%; adj.HR=2.86, 95%CI: 1.38–5.90) at 12 months after adjusted for potential covariates. We also observed that a higher level of glucose-to-HbA1c ratio was associated with an elevated risk of stroke recurrence and death.

**Conclusion:** Stress hyperglycemia, measured by glucose-to-HbA1c ratio, was related to an elevated risk of stroke recurrence and all-cause death in patients with acute ischemic stroke but without diabetes.

## Introduction

Stress hyperglycemia is different from various forms of diabetes mellitus and is common after suffering an acute illness such as myocardial infarction or stroke, even in the absence of preexisting diabetes (1–3). Some studies (3), but not all (4), have demonstrated that stress hyperglycemia appeared to be a marker of increased risk of short-term mortality and poor functional outcome after ischemic stroke in non-diabetic patients. Therefore, the association between stress hyperglycemia and outcome of stroke was still controversial. Additionally, stress hyperglycemia was defined according to absolutely but not relatively increased fasting or random glucose in non-diabetic patients in most previous studies (3, 4).

Glycated hemoglobin (HbA1c) is a well-validated measure of mean glucose concentration over ~2–3 months, representing the mean level of blood glucose before the onset of acute ischemic stroke event (5). Thus, the ratio of acute fasting plasma glucose (FPG) to HbA1c means the relative hyperglycemia, namely stress hyperglycemia. Recent studies showed that relative hyperglycemia, defined as glucose-to-HbA1c ratio (6), or defined as admission glucose divided by estimated average glucose derived from HbA1c (2, 7), might be a better predicter of outcomes of critical illness than absolute hyperglycemia.

We aimed to explore the relationship between stress hyperglycemia, which was measured by glucose-to-HbA1c ratio, and the outcome of non-diabetic patients with acute ischemic stroke in a prospective stroke registry study in China.

## Methods

### Study Participants

We used data from the Abnormal Glucose Regulation in Patients with Acute Stroke across China (ACROSS-China) registry. ACROSS-China registry is a nationwide, prospective hospital-based registry study and the details on the rationale, study design and main results were published previously (8). The main objective of the ACROSS-China registry is to investigate the relationship between abnormal glucose regulation and the outcome of stroke patients within 14 days after the onset of stroke events from May 2008 to December 2009 in China. The protocol of the study was approved by the ethics committee of Beijing Tiantan Hospital and other participating centers. We obtained written informed consents from all patients or his/her representatives before enrollment. In this analysis, we only included those patients with acute ischemic stroke but without a history of diabetes.

Acute ischemic stroke was diagnosed by the World Health Organization criteria (9) and confirmed by brain computed tomography (CT) or magnetic resonance imaging (MRI). The severity of stroke was assessed by the National Institutes of Health Stroke Scale (NIHSS) on admission. We classified all ischemic stroke based on the Trial of ORG 10172 in Acute Stroke Treatment (TOAST) criteria (10) as large artery atherosclerosis, small artery occlusion, cardio embolism, other, and undetermined subtypes, respectively.

### Data Collection

We collected baseline data on patient demographics, risk factors (blood pressure and smoking status), medical history (diabetes, hypertension, dyslipidemia, coronary heart disease, and atrial fibrillation), complications (pulmonary or urinary infection) and medication use during hospitalization for all patients. The data collections were performed through face-to-face interviews by trained research coordinators (all were neurologists) from participating centers and performed based on a standardized protocol. History of diabetes mellitus was defined as any self-reported history of diabetes mellitus and any use of anti-diabetic drugs before the symptom onset of stroke. All the historical and new observed atrial fibrillations were collected and further confirmed by at least one electrocardiogram.

### Assessment of Stress Hyperglycemia

The fasting venous blood samples within 2 days after hospitalization were drawn during the morning hours (range: 07:00–11:00) after an overnight fast (at least 8 h) to measure FPG and HbA1c. FPG was measured with an enzymatic method and HbA1c was tested using high-performance liquid chromatographic analysis (11). The laboratory personnel that performed the blood measurements were blinded to the patient's baseline characteristics and outcomes.

Stress hyperglycemia was estimated by the index of glucose-to-HbA1c ratio. We used the following formula to calculate glucose-to-HbA1c ratio: glucose-to-HbA1c ratio = FPG (mmol/L) / HbA1c (%). According to the quartiles of glucose-to-HbA1c ratio, the patients were further categorized into four even groups. Glucose-to-HbA1c ratio quantifies the extent of acute increase in blood glucose based on the baseline blood glucose levels.

### Patient Follow-Up and Outcomes Assessment

We assessed the outcomes of all patients through centralized telephone interviews by trained research coordinators (all were neurologists) at the participating hospitals. The outcomes of this study included stroke recurrence and all-cause death at 12 months. Stroke recurrence was defined as a new neurological deficit or re-hospitalization with a diagnosis of ischemic or hemorrhagic stroke (12, 13). Stroke recurrences associated with re-hospitalization were confirmed by the medical records from the attended hospitals. Case fatality was sourced to and confirmed on a death certificate from either the attended hospital or local citizen registry.

### Statistical Analysis

Categorical variables were presented as frequencies and percentages, while continuous variables were presented as mean with standard deviation or median with interquartile. The baseline characteristics of patients were compared among included and excluded patients, and among quartiles of glucose-to-HbA1c ratio using χ^2^ test for categorical variables (such as gender and medical histories) and ANOVA or the Kruskal-Wallis test for continuous variables (such as age and HbA1c).

The relationship of the categories of glucose-to-HbA1c ratio and the time to the first stroke recurrence and death were evaluated by proportional hazard Cox regression models with the lowest quartile as the reference. Through proportional hazard Cox regression models, we estimated adjusted hazard ratios (HRs) with their 95% confidence intervals (CIs) for each category of glucose-to-HbA1c ratio. We included a time-dependent covariate with interaction of the glucose-to-HbA1c ratio and a logarithmic function of survival time in the model to test the proportional hazard assumption. We performed three multivariate models to evaluate the association between glucose-to-HbA1c ratio and each outcome. In model 1, we only adjusted for age, gender, stroke severity, stroke subtype, and risk factors, including history of hypertension, history of hyperlipidemia, history of coronary heart disease and atrial fibrillation, and smoking status. In model 2, we additionally adjusted for complications during hospitalization, including pulmonary infection, and urinary infection. In model 3, we further adjusted for medicine use during hospitalization, including statins, thrombolytic, antiplatelet, antihypertensive, and anticoagulation agents.

Furthermore, we performed Cox regression models with restricted cubic splines for the glucose-to-HbA1c ratio (continuous variable) with adjustments for all potential covariates (model 3) to the pattern and magnitude of associations between the glucose-to-HbA1c ratio and the risk of stroke recurrence or death. The 25th percentile of the index of glucose-to-HbA1c ratio was used as the reference and the 5th, 25th, 50th, 75th, and 95th percentiles of the index of glucose-to-HbA1c ratio were used as the 5 knots for spline.

All analyses were performed by SAS software version 9.4 (SAS Institute Inc, Cary, NC). Two-sided *p* < 0.05 was considered as statistically significant.

## Results

### Study Participants

**Figure 1:**
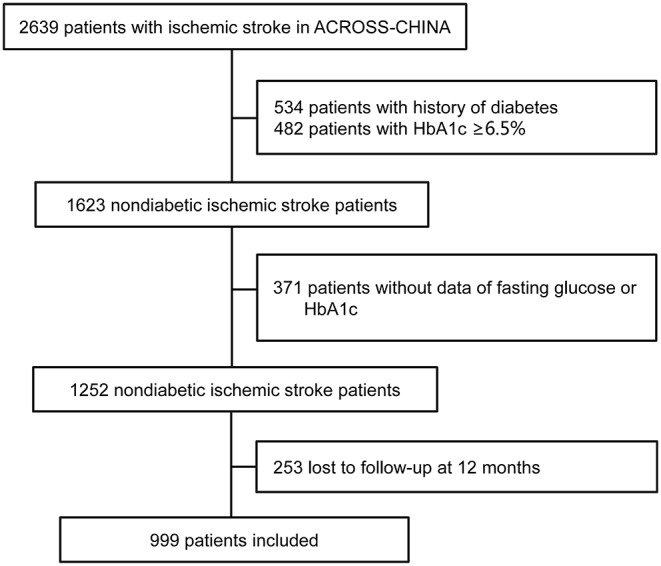
Flow diagram of the patient selection.

The study enrolled a total of 1,623 consecutive non-diabetic patients with acute ischemic stroke. After excluding patients without a test of FPG or HbA1c, or those lost to follow-up at 12 months, a total of 999 (61.6%) patients were included in this analysis (Figure 1). Table 1 shows the baseline demography and disease characteristics of the patients included in and those excluded from this analysis. According to Table 1, the patients included and excluded were well-balanced except that those excluded for missing data of glucose had more stroke subtype of cardio embolism and those excluded for loss to follow-up had less stroke subtype of large artery atherosclerosis. The median age of the included 999 patients was 61.7 (range 19–92), among which 354 (35.6%) patients were female.

**Table 1:** Baseline characteristics of patients included and excluded.

**Characteristics**	**Included (*n* = 999)**	**Excluded for missing glucose or HbA1c (*n* = 371)**	**Excluded for loss to follow-up (*n* = 253)**	***p***
Gender (female), *n* (%)	354 (35.td)	111 (30.1)	81 (32.4)	0.14
Age (year), mean (SD)	61.7 ± 13.0	62.2 ± 13.8	62.3 ± 13.1	0.55
History of hypertension, *n* (%)	586 (58.7)	223 (60.1)	145 (57.3)	0.78
History of hyperlipidemia, *n* (%)	106 (10.6)	29 (7.8)	21 (8.3)	0.22
History of atrial fibrillation, *n* (%)	61 (6.1)	35 (9.4)	16 (6.3)	0.09
History of coronary heart disease, *n* (%)	111 (11.1)	49 (13.2)	21 (8.3)	0.16
Smoking, *n* (%)				0.29
Non-smoker	563 (56.4)	224 (60.4)	143 (56.5)	
Ever smoker	90 (9.0)	41 (11.1)	25 (9.9)	
Current smoker	346 (34.6)	106 (28.6)	85 (33.6)	
NIHSS score on admission, median (IQR)	4 (2–8)	4 (2–8)	4.5 (2–8.5)	0.79
TOAST subtypes, *n* (%)				0.001
Cardio embolism	63 (6.3)	36 (9.7)	16 (6.3)	
Large artery atherosclerosis	624 (62.5)	238 (64.2)	147 (58.1)	
Small artery occlusion	256 (25.6)	67 (18.1)	62 (24.5)	
Other/undetermined	22 (2.2)	18 (4.9)	11 (4.3)	
Undefined	34 (3.4)	12 (3.2)	17 (6.7)	

*IQR, indicates interquartile range; NIHSS, National Institutes of Health stroke scale; SD, standard deviation; and TOAST, Trial of Org 10172 in Acute Stroke Treatment*.

Among the included patients, the median glucose-to-HbA1c ratio was 0.93 (interquartile: 0.83–1.07). The baseline demography and diseases characteristics of the patients by quartiles of glucose-to-HbA1c ratio were showed in Table 2. Patients with elevated level of glucose-to-HbA1c ratio were more likely to be non-smoker, have higher severity of stroke, higher proportion of pulmonary, and urinary infection. Additionally, patients with elevated level of glucose-to-HbA1c ratio had higher level of FPG (median FPG: 4.4, 5.0, 5.4, and 6.1 mmol/L in those with the 1st, 2nd, 3rd, and 4th quartile of glucose-to-HbA1c ratio) but lower level of HbA1c (median HbA1c: 5.9, 5.7, 5.5, and 5.0% in those with the 1st, 2nd, 3rd, and 4^th^ quartile of glucose-to-HbA1c ratio).

**Table 2:** Baseline characteristics according to the stress hyperglycemia states measured by glucose-to-HbA1c ratio.

	**Glucose-to-HbA1c ratio**				***p* value**
	Q1 (*n* = 257)^*^	**Q2 (*n* = 243)**	**Q3 (*n* = 253)**	**Q4 (*n* = 246)**	
Gender (female), *n* (%)	88 (34.5)	89 (36.9)	77 (30.6)	100 (40.7)	0.12
Age (year), mean (SD)	61.7 ± 13.9	63.1 ± 12.7	61.3 ± 12.6	60.6 ± 12.6	0.17
Systolic blood pressure (mm Hg), mean (SD)	143.4 ± 19.1	147.4 ± 22.9	147.2 ± 20.7	148.5 ± 21.2	0.06
Diastolic blood pressure (mm Hg), mean (SD)	85.0 ± 11.4	86.4 ± 12.5	86.3 ± 12.1	87.1 ± 12.4	0.49
History of hypertension, *n* (%)	153 (59.5)	138 (56.8)	153 (60.5)	142 (57.7)	0.83
History of hyperlipidemia, *n* (%)	29 (11.3)	27 (11.1)	27 (10.7)	23 (9.3)	0.90
History of atrial fibrillation, *n* (%)	16 (6.2)	12 (4.9)	15 (5.9)	18 (7.3)	0.75
History of coronary heart disease, *n* (%)	26 (10.1)	24 (9.9)	30 (11.9)	31 (12.6)	0.72
Smoking, *n* (%)					0.01
Non-smoker	132 (51.4)	136 (56.0)	134 (53.0)	161 (65.4)	
Previous smoker	19 (7.4)	27 (11.1)	26 (10.3)	18 (7.3)	
Current smoker	106 (41.2)	80 (32.9)	93 (36.8)	67 (27.2)	
Medicine use during hospitalization, *n* (%)					
Intravenous alteplase	7 (2.7)	9 (3.7)	11 (4.3)	6 (2.4)	0.61
Antiplatelet	167 (65.0)	157 (64.6)	158 (62.5)	156 (63.4)	0.93
Anticoagulation	9 (3.5)	13 (5.3)	14 (5.5)	16 (6.5)	0.49
Antihypertensive drugs	110 (42.8)	107 (44.0)	116 (45.8)	97 (39.4)	0.53
Diuretics	5 (1.9)	2 (0.8)	10 (4.0)	7 (2.8)	0.13
Beta blockers	9 (3.5)	8 (3.3)	11 (4.3)	9 (3.7)	0.93
Statin	144 (56.0)	124 (51.0)	124 (49.0)	109 (44.3)	0.07
Oral hypoglycemic agents	12 (4.7)	10 (4.1)	7 (2.8)	21 (8.5)	0.02
Insulin	3 (1.2)	2 (0.8)	3 (1.2)	13 (5.3)	0.001
Pulmonary infection, *n* (%)	14 (5.4)	12 (4.9)	22 (8.7)	36 (14.6)	<0.001
Urinary infection, *n* (%)	2 (0.8)	7 (2.9)	15 (5.9)	16 (6.5)	0.003
NIHSS score on admission, median (IQR)	4 (2–7)	4 (2–7)	4 (2–8)	6 (3–11)	<0.001
TOAST subtypes, *n* (%)					0.92
Cardio embolism	16 (6.2)	11 (4.5)	19 (7.5)	17 (6.9)	
Large artery atherosclerosis	164 (63.8)	149 (61.3)	155(61.3)	156(63.4)	
Small artery occlusion	62 (24.1)	72 (29.6)	65(25.7)	57(23.2)	
Other/undetermined	7 (2.7)	4 (1.6)	5(2.0)	6(2.4)	
Undefined	8 (3.1)	7 (2.9)	9(3.6)	10(4.1)	
FPG (mmol/L), median (IQR)	4.4 (4.1–4.7)	5.0 (4.7–5.3)	5.4 (5.0–5.8)	6.1 (5.5–7.0)	<0.001
HbA1c (%), median (IQR)	5.9 (5.6–6.2)	5.7 (5.4–6.0)	5.5 (5.1–5.9)	5.0 (4.5–5.6)	<0.001
Glucose-to-HbA1c ratio, median (IQR)	0.8 (0.7–0.8)	0.9 (0.9–0.9)	1.0 (1.0–1.0)	1.2 (1.1–1.3)	

*FPG, indicates fasting plasma glucose; HbA1c, glycated hemoglobin; IQR, interquartile range; NIHSS, National Institutes of Health stroke scale; SD, standard deviation; and TOAST, Trial of Org 10172 in Acute Stroke Treatment*.

* *Quartiles of glucose-to-HbA1c ratio, Q1 = ≤ 0.82, Q2 = 0.83–0.92, Q3 = 0.93–1.06, Q4 = ≥1.07*.

### Relationship Between Stress Hyperglycemia and Outcome

There were 105 (10.9%) recurrent stroke and 76 (7.6%) death at 12 months. Table 3 shows the 12-month outcomes after an ischemic stroke across quartiles of glucose-to-HbA1c ratio. Patients in the higher glucose-to-HbA1c ratio were associated with an elevated risk of stroke recurrence and death at 12 months (p for trend = 0.006 and 0.02, respectively). In the model with adjustment for patients demographics, stroke severity, stroke subtype, and vascular risk factors (model 1) and the model with further adjustment for complications (model 2), we found that patients with the highest quartile of glucose-to-HbA1c ratio was associated with an elevated risk of 12-month stroke recurrence and death. After further adjustment of medicine use during hospitalization (model 3), the highest quartile of glucose-to-HbA1c ratio remained to be associated with an elevated risk of 12-month stroke recurrence (16.0 vs. 8.3%; adj.HR = 2.19, 95%CI: 1.26–3.83) and death (13.0% vs. 4.3%; adj.HR=2.86, 95%CI: 1.38–5.90) compared with patients with the lowest quartile. All the assumptions of proportional hazards for Cox regression were met (*p* = 0.40 for the outcome of stroke recurrence and *p* = 0.10 for the outcome of death).

**Table 3:** Adjusted hazard ratios of outcomes at 12 months according to glucose-to-HbA1c ratio quartiles.

**Outcomes**	**Glucose-to-HbA1c groups**	***n***	**Events, *n* (%)**	Model 1^*^		**Model 2^†^**		**Model 3^‡^**	
				**HR (95%CI)**	***p* value**	**HR (95%CI)**	***p* value**	**HR (95%CI)**	**p value**
Stroke recurrence	Q1 (≤0.82)	254	21 (8.3)	Ref.		Ref.		Ref.	
	Q2 (0.83–0.92)	236	23 (9.7)	1.13 (0.62–2.04)	0.69	1.14 (0.63–2.06)	0.67	1.16 (0.64–2.10)	0.64
	Q3 (0.93–1.06)	346	24 (9.8)	1.22 (0.68–2.19)	0.52	1.25 (0.69–2.25)	0.46	1.26 (0.70–2.27)	0.45
	Q4 (≥1.07)	231	37 (16.0)	2.09 (1.20–3.65)	0.009	2.18 (1.25–3.80)	0.006	2.19 (1.26–3.83)	0.006
	p for trend				0.009		0.006		0.006
Death	Q1 (≤0.82)	257	11 (4.3)	Ref.		Ref.		Ref.	
	Q2 (0.83–0.92)	243	19 (7.8)	2.08 (0.98–4.40)	0.06	2.06 (0.96–4.42)	0.07	2.05 (0.96–4.38)	0.07
	Q3 (0.93–1.06)	253	14 (5.5)	1.36 (0.62–3.02)	0.44	1.33 (0.60–2.96)	0.49	1.31 (0.59–2.93)	0.51
	Q4 (≥1.07)	246	32 (13.0)	2.68 (1.32–5.44)	0.006	2.81 (1.36–5.79)	0.005	2.86 (1.38–5.90)	0.005
	p for trend				0.02		0.02		0.02

*CI indicates confidence interval; HbA1c, glycated hemoglobin; and HR, hazard ratio*.

* *Model 1: adjusted for age, gender, National Institutes of Health stroke score, stroke subtype, history of hypertension, hyperlipidemia, coronary heart disease, atrial fibrillation, and smoking status*.

† *Model 2: adjusted for Model 1 + pulmonary infection and urinary infection during hospitalization*.

‡ *Model 3: adjusted for Model 2 + statins, thrombolytic, antiplatelet, antihypertensive, and anticoagulation agents use during hospitalization*.

**Figure 2:**
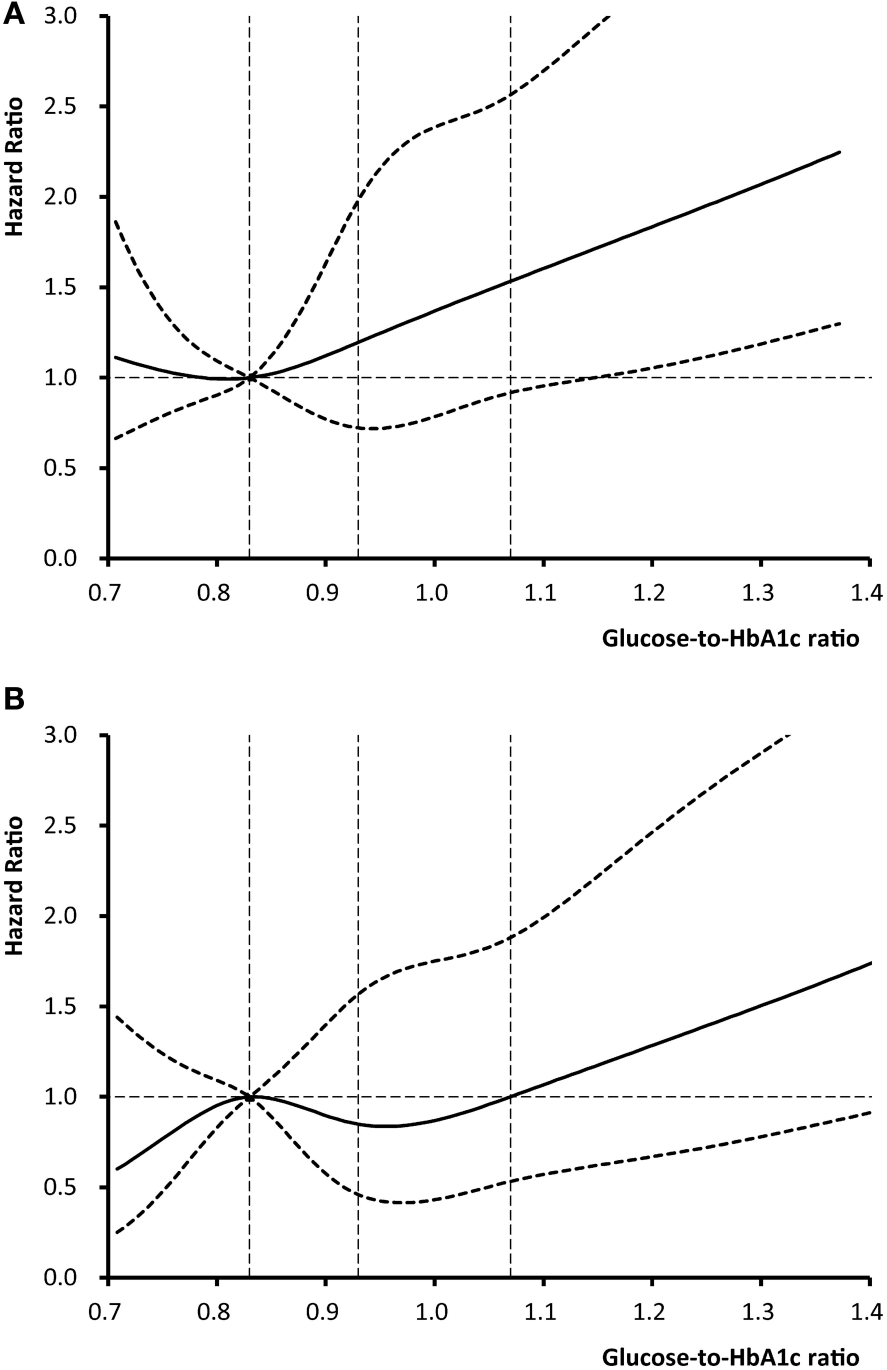
Adjusted hazard ratios of **(A)** stroke recurrence and **(B)** death according to glucose-to-HbA1c ratio. The solid line indicates adjusted hazard ratio and the dashed lines the 95% confidence interval bands. Reference is the 25th percentile of glucose-to-HbA1c ratio (0.83). The vertical dashed lines indicate the 1st, 2nd, and 3rd quartiles of glucose-to-HbA1c ratio. Data were fitted using a Cox regression model of restricted cubic spline with 5 knots (the 5th, 25th, 50th, 75th, 95th percentiles) for glucose-to-HbA1c ratio, adjusting for potential covariates. The lowest 5% and highest 5% of patients were not shown in the figures for small sample sizes.

Figure 2 shows the non-linear relationship between level of glucose-to-HbA1c ratio and the risk of outcome events using Cox regression models with restricted cubic spline. We observed that higher level of glucose-to-HbA1c ratio was associated with an elevated risk of 12-month stroke recurrence and death.

## Discussion

In this nationwide prospective registry study in China, we found that the stress hyperglycemia on admission measured by glucose-to-HbA1c ratio was associated with an elevated risk of stroke recurrence and mortality at 12 months in non-diabetic patients with acute ischemic stroke. Higher level of glucose-to-HbA1c ratio was associated with an elevated risk of poor prognosis of stroke.

There is a high prevalence of stress hyperglycemia in non-diabetic patients with acute illness like stroke, ranging from 8 to 63% based on variable study population and measurement of stress hyperglycemia (3). Several previous studies identified the stress hyperglycemia as a marker to predict higher risk of in-hospital mortality in patients with myocardial infarction (14), and higher risk of in-hospital mortality (3), 3-month poor functional outcome (15) and 3-month mortality (16) in non-diabetic patients with acute ischemic stroke. However, a Greece study showed that the association between stress hyperglycemia and in-hospital mortality after acute ischemic stroke did not persist after adjustment for stroke severity (4). Furthermore, these previous studies mostly measured stress hyperglycemia based on absolute increase of glycaemia with absence of diabetes. Our previous study showed that stress hyperglycemia, measured by the index of glucose-to-glycated albumin (GA) ratio, another relative measure of stress hyperglycemia, was associated with an elevated risk of 3-month new occurrence of stroke in patients with acute minor ischemic stroke or transient ischemic attack. The present study validated the association of stress hyperglycemia and long-term (12-month) stroke recurrence in a large-scale prospective stroke registry, and further added evidence that stress hyperglycemia was associated with higher risk of long-term (12 months) mortality in non-diabetic patients with acute ischemic stroke.

The mechanisms underlying the relationship between stress hyperglycemia and poor prognoses of stroke are not fully known. There were several explanations to understand the association. First, stress hyperglycemia may be a marker that represents the extent of ischemic damage after stroke. Stress hyperglycemia is relative rapid increase of blood glucose resulted from the neuro-hormonal derangements and inflammatory response that occur following the acute critical events like acute stroke (1, 2). Stress hyperglycemia is caused by high hepatic output of glucose resulting from a complicated interaction of hormones such as catecholamines, cortisol, and cytokines (1). Patients with severe or fatal strokes might develop stress hyperglycemia due to high level release of these inflammatory and vasoconstrictive factors (3). Second, hyperglycemia may be directly neurotoxic to the ischemic penumbra and caused more neurons to be injured and unviable (17–19). Third, oscillating glucose may aggravate injure of endothelial function and oxidative stress, which are two key factors that cause vascular events (20, 21). Fourth, hyperglycemic patients are relatively deficient in insulin (3). Our previous studies showed that insulin resistance and β-cell dysfunction were related to an elevated risk of poor outcome of non-diabetic patients with ischemic stroke (12, 13). Finally, hyperglycemia may have a deleterious effect on platelet function mediation by superoxide production and induce platelet aggregation (22). These pathological changes may result in a poor prognosis of stroke and tend to promote atherosclerosis and subsequent stroke. Our study also showed that the association between glucose-to-HbA1c ratio and outcome followed a threshold effect with an increased risk only in the highest quartile, indicating that sharp stress hyperglycemia may have more deleterious effect and result in a worse stroke outcome. The mechanism of underlining the association between stress hypoglycemia and poorer stroke outcome needs further investigation in the future. This also implicated that stress hyperglycemia was a potential treatment target to improve stroke outcome. However, this needs further investigation in well-designed randomized control trials.

In most of the previous studies, stress hyperglycemia was defined as absolute hyperglycemia without evidence of previously diagnosed diabetes or deterioration of pre-illness glycemic control with pre-existing diabetes (1, 3). But this diagnostic criterion did not consider the background glucose level and could not differentiate the stress hyperglycemia from diabetes. On the contrary, glucose-to-HbA1c ratio, the indicator we used to measure stress hyperglycemia, reflects a quantitative measurement of the relative acute rapid increase of blood glucose level based on glucose level prior stroke. Recent studies demonstrated that relative hyperglycemia, defined as glucose-to-HbA1c ratio (6) or defined as admission glucose level divided by estimated average blood glucose level obtained by regression with HbA1c (2), might be a better predicter for outcomes of critical diseases than absolute hyperglycemia. Furthermore, unlike glucose-to-GA ratio, another relative measure of stress hyperglycemia, measures based on HbA1c could be commonly used and generalized in the clinical practice. Glucose-to-HbA1c ratio could be more stable than glucose-to-GA ratio since HbA1c reflects mean glycaemia over ~2–3 months while GA only represents mean glycaemia over ~2–4 weeks. On the other hand, measures of stress hyperglycemia based on HbA1c required a separate specimen of whole blood and may be affected by chronic kidney disease, anemia, or hemoglobinopathies (23). Nevertheless, HbA1c is a well-established biomarker and is commonly used in the clinical practice. Our study has suggested an easy-to-perform approach to detect and quantify stress hyperglycemia. This could be of importance to predict the outcome of non-diabetic patients with ischemic stroke at the acute phage.

Several limitations should be acknowledged when explaining these results. First, about one third of patients were excluded due to failure to test fasting glucose or HbA1c, or loss to follow-up in this study. In fact, the baseline demography and disease characteristics were well-balanced among patients included in this analysis and those excluded, except that those included in the analysis had lower proportion of history of atrial fibrillation and stroke subtype of cardio embolism. Second, our study may have selection bias since only 35.6% of patients were female, median NIHSS scores are low (median score = 4), only 6.3% of patients had a cardio-embolic stroke and over 62.5% of patients had large-artery atherosclerosis. This may limit the generalization of our findings. Third, the generalizability of the results may be limited because only Chinese patients were enrolled in this study. The Chinese patients have a higher prevalence of intracranial atherosclerosis than the Western population (24, 25).

In conclusion, in this largescale stroke registry, stress hyperglycemia, measured by glucose-to-HbA1c ratio, was associated with an elevated risk of 12-month stroke recurrence and mortality in non-diabetic patients with acute ischemic stroke. Our findings may help doctors to assess the outcome of ischemic stroke at the acute phage in clinical practice.

## Data Availability

The datasets generated for this study are available on request to the corresponding author.

## Ethics Statement

The studies involving human participants were reviewed and approved by the ethics committee of Beijing Tiantan Hospital and all participating sites. The patients and participants provided their written informed consent to participate in this study.

## Author Contributions

BZ: study concept and design, analysis and interpretation of data, drafting of the manuscript. YP: acquisition of data, drafting of the manuscript. JJ, XM, and LL: acquisition of data. XZ: acquisition of data, study supervision or coordination. YiW: study concept and design, acquisition of data, analysis and interpretation of data. YoW: study concept and design, obtaining funding, analysis and interpretation of data. ZW: study concept and design, acquisition of data, study supervision or coordination.

### Conflict of Interest Statement

The authors declare that the research was conducted in the absence of any commercial or financial relationships that could be construed as a potential conflict of interest.

## References

[1] Dungan KM Braithwaite SS Preiser JC. Stress hyperglycaemia. Lancet. (2009) 373:1798–807. 10.1016/S0140-6736(09)60553-5 19465235 PMC3144755

[2] Roberts GW Quinn SJ Valentine N Alhawassi T O'Dea H Stranks SN. Relative hyperglycemia, a marker of critical illness: introducing the stress hyperglycemia ratio. J Clin Endocrinol Metab. (2015) 100:4490–7. 10.1210/jc.2015-2660 26485219

[3] Capes SE Hunt D Malmberg K Pathak P Gerstein HC. Stress hyperglycemia and prognosis of stroke in nondiabetic and diabetic patients: a systematic overview. Stroke. (2001) 32:2426–32. 10.1161/hs1001.096194 11588337

[4] Tziomalos K Dimitriou P Bouziana SD Spanou M Kostaki S Angelopoulou SM. Stress hyperglycemia and acute ischemic stroke in-hospital outcome. Metabolism. (2017) 67:99–105. 10.1016/j.metabol.2016.11.011 28081783

[5] Emerging Risk Factors C Di Angelantonio E Gao P Khan H Butterworth AS Wormser D Glycated hemoglobin measurement and prediction of cardiovascular disease. JAMA. (2014) 311:1225–33. 10.1001/jama.2014.1873 24668104 PMC4386007

[6] Su YW Hsu CY Guo YW Chen HS. Usefulness of the plasma glucose concentration-to-HbA1c ratio in predicting clinical outcomes during acute illness with extreme hyperglycaemia. Diabetes Metab. (2017) 43:40–7. 10.1016/j.diabet.2016.07.036 27663631

[7] Chen X Liu Z Miao J Zheng W Yang Q Ye X. High stress hyperglycemia ratio predicts poor outcome after mechanical thrombectomy for ischemic stroke. J Stroke Cerebrovasc Dis. (2019) 28:1668–73. 10.1016/j.jstrokecerebrovasdis.2019.02.022 30890395

[8] Jia Q Zheng H Zhao X Wang C Liu G Wang Y. Abnormal glucose regulation in patients with acute stroke across China: prevalence and baseline patient characteristics. Stroke. (2012) 43:650–7. 10.1161/STROKEAHA.111.633784 22267822

[9] Stroke 1989 Recommendations on stroke prevention, diagnosis, and therapy. Report of the WHO Task Force on Stroke and other Cerebrovascular Disorders. Stroke. (1989) 20:1407–31. 10.1161/01.STR.20.10.1407 2799873

[10] Adams HP Jr Bendixen BH Kappelle LJ Biller J Love BB Gordon DL. Classification of subtype of acute ischemic stroke. Definitions for use in a multicenter clinical trial. TOAST. Trial of Org 10172 in acute stroke treatment. Stroke. (1993) 24:35–41. 10.1161/01.STR.24.1.35 7678184

[11] Cushman M Cornell ES Howard PR Bovill EG Tracy RP. Laboratory methods and quality assurance in the Cardiovascular Health Study. Clin Chem. (1995) 41:264–70. 7874780

[12] Pan Y Chen W Jing J Zheng H Jia Q Li H. Pancreatic beta-cell function and prognosis of nondiabetic patients with ischemic stroke. Stroke. (2017) 48:2999–3005. 10.1161/STROKEAHA.117.018203 28954919

[13] Jing J Pan Y Zhao X Zheng H Jia Q Mi D. Insulin resistance and prognosis of nondiabetic patients with ischemic stroke: the ACROSS-china study. (Abnormal Glucose Regulation in Patients With Acute Stroke Across China). Stroke. (2017) 48:887–93. 10.1161/STROKEAHA.116.015613 28235959

[14] Capes SE Hunt D Malmberg K Gerstein HC. Stress hyperglycaemia and increased risk of death after myocardial infarction in patients with and without diabetes: a systematic overview. Lancet. (2000) 355:773–8. 10.1016/S0140-6736(99)08415-9 10711923

[15] Marulaiah SK Reddy MP Basavegowda M Ramaswamy P Adarsh LS. Admission hyperglycemia an independent predictor of outcome in acute ischemic stroke: a longitudinal study from a tertiary care hospital in South India. Niger J Clin Pract. (2017) 20:573–80. 10.4103/1119-3077.206368 28513516

[16] Roquer J Giralt-Steinhauer E Cerda G Rodriguez-Campello A Cuadrado-Godia E Jimenez-Conde J. Glycated hemoglobin value combined with initial glucose levels for evaluating mortality risk in patients with ischemic stroke. Cerebrovasc Dis. (2015) 40:244–50. 10.1159/000440735 26484656

[17] Rosso C Pires C Corvol JC Baronnet F Crozier S Leger A. Hyperglycaemia, insulin therapy and critical penumbral regions for prognosis in acute stroke: further insights from the INSULINFARCT trial. PLoS ONE. (2015) 10:e0120230. 10.1371/journal.pone.0120230 25793765 PMC4368038

[18] Parsons MW Barber PA Desmond PM Baird TA Darby DG Byrnes G. Acute hyperglycemia adversely affects stroke outcome: a magnetic resonance imaging and spectroscopy study. Ann Neurol. (2002) 52:20–8. 10.1002/ana.10241 12112043

[19] Luitse MJ van Seeters T Horsch AD Kool HA Velthuis BK Kappelle LJ. Admission hyperglycaemia and cerebral perfusion deficits in acute ischaemic stroke. Cerebrovasc Dis. (2013) 35:163–7. 10.1159/000346588 23429063

[20] Ceriello A Esposito K Piconi L Ihnat MA Thorpe JE Testa R. Oscillating glucose is more deleterious to endothelial function and oxidative stress than mean glucose in normal and type 2 diabetic patients. Diabetes. (2008) 57:1349–54. 10.2337/db08-0063 18299315

[21] Monnier L Mas E Ginet C Michel F Villon L Cristol JP. Activation of oxidative stress by acute glucose fluctuations compared with sustained chronic hyperglycemia in patients with type 2 diabetes. JAMA. (2006) 295:1681–7. 10.1001/jama.295.14.1681 16609090

[22] Worthley MI Holmes AS Willoughby SR Kucia AM Heresztyn T Stewart S. The deleterious effects of hyperglycemia on platelet function in diabetic patients with acute coronary syndromes mediation by superoxide production, resolution with intensive insulin administration. J Am Coll Cardiol. (2007) 49:304–10. 10.1016/j.jacc.2006.08.053 17239711

[23] Yazdanpanah S Rabiee M Tahriri M Abdolrahim M Rajab A Jazayeri HE. Evaluation of glycated albumin. (GA) and GA/HbA1c ratio for diagnosis of diabetes and glycemic control: a comprehensive review. Crit Rev Clin Lab Sci. (2017) 54:219–32. 10.1080/10408363.2017.1299684 28393586

[24] Suri MF Qiao Y Ma X Guallar E Zhou J Zhang Y. Prevalence of intracranial atherosclerotic stenosis using high-resolution magnetic resonance angiography in the general population: the atherosclerosis risk in communities study. Stroke. (2016) 47:1187–93. 10.1161/STROKEAHA.115.011292 27056984 PMC5319392

[25] Wang Y Zhao X Liu L Soo YO Pu Y Pan Y. Prevalence and outcomes of symptomatic intracranial large artery stenoses and occlusions in China: the Chinese Intracranial Atherosclerosis. (CICAS) Study. Stroke. (2014) 45:663–9. 10.1161/STROKEAHA.113.003508 24481975

